# Identification of Critical Region Responsible for Split Hand/Foot Malformation Type 3 (SHFM3) Phenotype through Systematic Review of Literature and Mapping of Breakpoints Using Microarray Data

**DOI:** 10.3390/microarrays5010002

**Published:** 2015-12-24

**Authors:** Catherine F. Li, Katie Angione, Jeff M. Milunsky

**Affiliations:** Center for Human Genetics, Cambridge, MA 02139, USA; cli@chginc.org (C.F.L.); kangione@chginc.org (K.A.)

**Keywords:** Split hand/foot Malformation (SHFM3), 10q24.31–q24.32, duplication, *FBXW4*, *BTRC*

## Abstract

Split hand/foot malformation (SHFM) is a limb malformation with underdeveloped or absent central digital rays, clefts of hands and feet, and variable syndactyly of the remaining digits. There are six types of SHFM. Here, we report a boy with SHFM type 3 having normal 4th and 5th digits, absent 2nd and 3rd digits, and a 4th finger flexion deformity, as well as absent 2nd, 3rd and 4th toes bilaterally. His father, two paternal uncles, and two paternal first cousins have similar phenotype. Chromosome analysis showed a normal male karyotype. A 514 kb gain at 10q24.31–q24.32 (chr10:102,962,134–103,476,346, hg19) was identified using 6.0 Single nucleotide polymorphism (SNP) microarray, resulting in the duplication of nine genes, including *BTRC* and *FBXW4*. A detailed systematic review of literature and mapping of breakpoints using microarray data from all reported cases in PubMed and DECIPHER were conducted, and exon 1 of *BTRC* gene was identified as the critical region responsible for the SHFM3 phenotype. The potential mechanism and future studies of this critical region causing the SHFM3 phenotype are discussed.

## 1. Introduction

Split-hand/foot malformation (SHFM) is a limb malformation with underdeveloped or absent central digital rays, clefts of hands and feet, and variable syndactyly of the remaining digits. It is a rare condition, affecting 1 in 8500–25,000 newborns. So far, six genetic loci have been identified for non-syndromic SHFM, and classified as SHFM 1 to SHFM6. SHFM1 is at 7q21 (OMIM 183600), SHFM2 at Xq26 (OMIM 313350), SHFM3 at 10q24 due to tandem duplication (OMIM 246560), SHFM4 at 3q27 due to mutations in *TP63* gene (OMIM 605289), SHFM5 at 2q31 (OMIM 606708), and SHFM6 at 12q13 due to mutations in *WNT10B* gene. Here, we report a boy with SHFM type 3, having 514 kb duplication at 10q24.31–q24.32 identified using 6.0 SNP microarray. A literature review of reported cases, mapping of breakpoints using microarray data to identify the critical region responsible for the SHFM3 phenotype, and discussion of the potential mechanism and future studies of this critical region causing the SHFM3 phenotype are presented below.

## 2. Results and Discussion

### 2.1. Chromosome Karyotyping and SNP Microarray Results for the Proband

**Figure 1:**
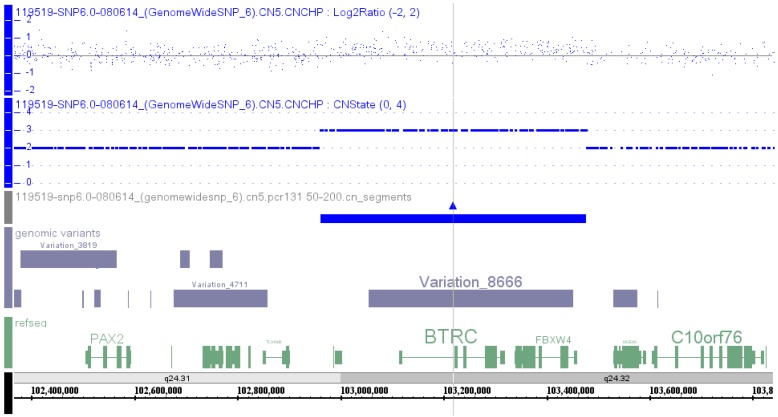
SNP microarray result in the proband: arr[hg19] 10q24.31q24.32(102,962,134-103,476,346)×3. Tracks were arranged from top to bottom showing Log2 ratio (blue dots), copy number state (blue dot line), segments with copy number gain (**blue**), genomic variants (**grey**) and genes in the region (**green**), as well as chromosome band and genomic coordinates. Gray line is the center of the region with copy number gain.

High resolution chromosome and 6.0 SNP microarray analyses were performed. The result showed a normal 46,XY karyotype and a 514 kb duplication at 10q24.31-10q24.32 (chr10:102,962,134–103,476,346, hg19) (Figure 1), containing *LBX1*, *FLJ41350*, *AX747408*, *BTRC*, *POLL*, *DPCD*, *MIR3158-1*, *MIR3158-2*, and *FBXW4* from centromeric to telomeric direction (Figure S1a, and Table S1, Patient 1). Fluorescence *in situ* hybridization (FISH) confirmation of the microarray aberration was not performed. Microarray and FISH results on parental and other affected family members were not available.

### 2.2. Breakpoint Mapping for SHFM3 Cases

#### 2.2.1. SHFM3 Cases Reported in PubMed

PubMed literature review on reported cases was conducted up to 23 July 2015, and there were 14 publications regarding duplication at 10q24 in SHFM3, including seven publications using microarray to define the boundary of the duplication. The genes and exons in the duplicated region are presented from centromeric to telomeric direction (Figure S1a, and Table S1).

Tandem duplication at 10q24 in patients with SHFM3 was first reported by de Mollerat *et al.* [1] in 2003 using Southern, pulsed field gel electrophoresis and dosage analyses. This duplicated region contained a disrupted extra copy of the *FBXW4* gene and the entire *LBX1* and *BTRC* genes, known to be involved in limb development. The smallest duplicated region (440 kb) defines the minimal duplicated region common to all patients, which narrows the number of genes included in the duplicated region to genes from *LBX1* to a portion of the *FBXW4* gene involving a region of about 440–570 kb (Table S1, Patient 26–32). The proximal and distal breakpoints were within a 130 and 80 kb region, respectively. The proximal breakpoints are located at the intergenic region centromeric to *LOC159673* (two cases), centromeric to *HUG1* (*TLX1NB1*) (one case), telomeric to *TBX1* (one case), telomeric to *LOC203696* (centromeric to *LBX1*) (three cases), respectively. Therefore, the majority of the proximal breakpoints are located at the intergenic region centromeric to *LBX1*. The distal breakpoints located at 5′ untranslated region (UTR) (two cases), intron 2 (one case) and intron 5 (four cases) of *FBXW4* gene, respectively. Therefore, the majority of the distal breakpoints are located at intron 5 of *FBXW4* gene.

To refine the minimum duplicated region and to further characterize the SHFM3 locus, Kano *et al.* [2] in 2005 screened 28 non-syndromic SHFM families for tandem genomic duplication of 10q24 by Southern blot and sequence analysis of the *FBXW4* gene. Of 28 families, only two showed genomic rearrangements. Representative patients from the two families exhibit typical SHFM phenotypes with symmetrically affected hands and feet. One patient is a familial case with a 512 kb tandem duplication containing genes from *LBX1* to *FBXW4* (Table S1, Patient 33), which is similar to the proband in this study. The other patient is a sporadic case arising from a *de novo*, 447 kb duplication of maternal origin, containing genes from *LBX1* to a portion of *FBXW4* (exon 9–6) (Table S1, Patient 34). Therefore, the proximal breakpoints in both cases are located at the intergenic region centromeric to *LBX1*, while the distal breakpoints are located at intron 5 of *FBXW4* gene in the smaller duplication case, and at 5′UTR of *FBXW4* gene in the longer duplication case.

Using FISH and genomic DNA quantitative PCR, Lyle *et al.* [3] in 2006 showed duplication at 10q24 locus in 12 patients with SHFM3, in which seven patients have one duplicated segment, while five patients have two discontinuous duplicated segments. The proximal and distal breakpoint clusters are maximally about 160 kb and about 60 kb, respectively. In patients with only one duplicated segment, the distal breakpoint is between exon 3 and 7 of *FBXW4* in all patients, while the proximal breakpoint is between *KAZALD1* exon 5 and *TLX1NB1* (*HUG1*) intron 2 in one patient, between *TLX1NB1* (*HUG1*) intron 2 and *LBX1* exon 1 in four patients including the daughter in the RK040 family, and from *LBX1* exon 1 to the intergenic region between *AX747408* and *BTRC* in two patients including the father in the RK040 family. Therefore, the proximal breakpoint changes when it transmits from the father to the daughter. The duplicated segment contains genes from *TLX1NB1* to a portion of *FBXW4* (exon 9–7) in one patient; from *LBX1* to a portion of *FBXW4* (exon 9–7) in four patients including the daughter in the RK040 family; from *BTRC* to a portion of *FBXW4* (exon 9–7) in two patients including the father in the RK040 family (Table S1, Patient 28, and 35 to 40).

In patients with two discontinuous duplicated segments, the centromeric duplicated segment contains *TLX1NB1* and the telomeric duplicated segment contains genes from *LBX1* to a portion of *FBXW4* (exon 9–7) in two patients; the centromeric duplicated segment contains *TLX1NB1* and *TLX1* and the telomeric duplicated segment contains genes from *BTRC* to a portion of *FBXW4* (exon 9–7) in two patients; the centromeric duplicated segment contains genes from *LBX1* to a portion of *FBXW4* (exon 9–3) and telomeric duplicated segment contains *FGF8* in one patient. For the centromeric duplicated region, the proximal breakpoint is between *KAZALD1* exon 5 and *TLX1NB1* intron 2, and the distal breakpoint is between *TLX1* exon 3 and *LBX1* exon 1 in four patients; the proximal breakpoint is between *TLX1* exon 3 and *LBX1* exon 1, and the distal breakpoint is between *FBXW4* exon 3 and *FGF8* exon 2 in one patient. For the telomeric duplicated region, the proximal breakpoint is between *TLX1* exon 3 and *LBX1* exon 1, and the distal breakpoint is between exon 3 and 7 of *FBXW4* in two patients; the proximal breakpoint is from *LBX1* exon 1 to the intergenic region between *AX747408* and *BTRC*, and the distal breakpoint is between exon 3 and 7 of *FBXW4* in two patients; the proximal breakpoint is between *FBXW4* exon 3 and *FGF8* exon 2, and the distal breakpoint is between *FGF8* exon 2 and *MGEA5* exon 2 in one patient (Table S1, Patient 26, and 29–32).

In addition, using RNA quantitative PCR, expression analysis of 13 candidate genes within and flanking the duplicated region showed that *BTRC* (present in three copies) and *SUFU* (present in two copies) were overexpressed in SHFM3 patients compared to controls, suggesting that SHFM3 may be caused by overexpression of *BTRC* and *SUFU*, both of which are involved in beta-catenin signaling [3].

Abnormal bands were observed using pulsed-field gel electrophoresis in eight cases, all but one being non-syndromic reported by Everman *et al.* in 2006 [4]. In all but one case, abnormal bands were observed with probes to the genes *FBXW4* and *BTRC* and to the centromeric region approximately 400-kb upstream of *FBXW4*. In the remaining case, abnormal bands were observed with probes to *FBXW4* and *BTRC*, but not with a probe to the centromeric region.

Array comparative genomic hybridization was first performed by Dimitrov *et al.* [5] in 2010 on seven individuals from four families with SHFM. In the first family, two siblings have distal limb deficiency with micrognathia syndrome (DLDMS). A 532.77 kb triplication at 10q24 was found in the more severely affected brother (Table S1, Patient 2), while duplication of the same region was present in the mildly affected sister (Table S1, Patient 3). This region contains genes from LBX1 to FBXW4. The patient in the second family with a *de novo* DLDMS has 528.72 kb duplication at 10q24. There are genes from *LBX1* to *FBXW4* in the duplicated region (Table S1, Patient 4). In the third family, a 597.29 kb duplication at 10q24 was found in the sister with SHFM, the brother with DLDMS, and shown as somatic mosaicism for this duplication in the phenotypically normal mother. The duplicated region contains genes from *LINC01514* to *FBXW4* (Table S1, Patient 5–7). The patient in the fourth family has syndromic SHFM, who has 658.43 kb duplication at 10q24, which contains genes from a portion of *TLX1NB* (exon 2–1, breakpoint at intron 2) to a portion of *FGF8* (exon 6–2, breakpoint at intron 1) (Table S1, Patient 8). FISH was performed in Patients 2, 3 and 5, while qPCR was conducted in all cases to confirm the array comparative genomic hybridization results.

Two affected brothers were found to have a small duplication of approximately 539 kb at 10q24.31-q24.32 through array-based comparative genomic hybridization by Filho *et al.* [6] in 2011. The duplicated region contains genes from a portion of *LINC01514* (exon 6, breakpoint at intron 5) to *FBXW4*. The patients’ sister and father do not have the duplication, but qPCR showed that the mother’s DNA carries the duplication in 20% of blood lymphocytes (Table S1, Patient 9–11). The microarray aberrations found in both affected brothers were confirmed by qPCR.

Dai *et al.* [7] in 2013 found the duplication at 10q24.31-q24.32 containing two discontinuous DNA fragments using Affymetrix cytogenetic 2.7M array in a SHFM family with four-generation-span. The proband (IV:3) was a three-year-old boy with severe distal deficiency affecting all four limbs. The other three patients exhibited similar phenotypes, although phenotypic variations were observed between affected family members. Triphalangeal thumb was only identified in the boy’s paternal grandfather (II:5), and complete 1/2 toe syndactyly in the boy (IV:3). Similar hypoplasia/agenesis of 1st ray existed in the boy, his father (III:9) and paternal aunt (III:10). No non-limb malformations were identified in any patient. The centromeric duplicated segment is 247 kb in the boy, 257 kb in the boy’s paternal grandfather and paternal aunt, and 259 kb in the boy’s father. The centromeric duplicated segment involves genes from *LINC01514* to a portion of *BTRC* (exon 1, breakpoint at intron 1) in all patients. The proximal breakpoint is at the same location for all patients, while the distal breakpoint is different among the boy, his father, and his paternal grandfather, although it is the same in the paternal grandfather and paternal aunt. Therefore, the distal breakpoint for the centromeric duplicated segment changes when it transmits from the father to the son, however, it stays the same when it transmits from the father to the daughter. The telomeric duplication is 114 kb in the boy and his paternal grandfather, 116 kb in his paternal aunt, and 125 kb in his father. The telomeric duplicated segment encompasses genes from *POLL* to a portion of *FBXW4* (exon 9–2, breakpoint at intron 1) in all patients. The distal breakpoint is at the same location for all patients, while the proximal breakpoint is different among his paternal grandfather, his father and his paternal aunt, but it is the same between the boy and his paternal grandfather. Therefore, the proximal breakpoint for the telomeric duplicated segment changes when it transmits from the father to both his son and daughter (Table S1, Patient 12–15). The microarray aberrations found in all cases were confirmed by qPCR.

A two-year-old boy having SHFM of all four limbs with the hands more severely affected than the feet was reported by Ockeloen *et al.* [8] in 2013. A 250K Affymetrix SNP array analysis showed a 600 kb gain at 10q24.31–q24.32. The duplicated region contains genes from a portion of *TLX1NB* (part of exon 3–1, breakpoint at exon 3) to a portion of *FBXW4* (exon 9–2, breakpoint at intron 1) (Table S1, Patient 16).

Affymetrix SNP 6.0 array was used to perform a genome-wide copy number variation scan, and quantitative real-time PCR (qPCR) was applied to validate the identified genomic duplication by Liu *et al.* [9] in 2014 in order to identify the potential pathogenic mutation in a Chinese family with SHFM. A microduplication of about 560 kb on the chromosome 10q24 was identified, and the qPCR assay confirmed the presence of this microduplication in all the available affected family members (Table S1, Patient 47).

To identify genomic aberrations underlying pathogenesis of SHFM in two Chinese families and to provide genetic counseling and prenatal diagnosis for them, Wang *et al.* [10] used array-based comparative genomic hybridization to analyze both blood and amniotic fluid samples from one of the families and showed a 662.3 kb duplication at 10q24.31–q24.32 (Table S1, Patient 48).

The most recent publication was from Chen *et al.* [11] in 2014 for a patient with SHFM using genome-wide copy number variation SNP microarray, and the tiny copy number variations were verified by real-time fluorescent quantitative PCR. The results of SNP microarray has revealed that the patient has carried a 394 kb duplication at 10q24.31–q24.32, which contains genes from *LBX1* to a portion of *DPCD* (exon 1, breakpoint at intron 1). By real-time fluorescent quantitative PCR, the duplicate area encompassing the pathogenic genes was verified, and duplication in exon 9 of the nearby *FBXW4* gene was detected (Table S1, Patient 17).

#### 2.2.2. SHFM3 Cases Reported in DECIPHER Database

Searching of DECIPHER database using SHFM3 as a keyword was performed on 23 July 2015, and there were 16 entries. Clinical information was available in 11 cases, and limb anomalies were present in nine cases. Seven patients had duplication at 10q24.31-q24.32. Clinical information was available in five patients, all having limb anomalies with classical SHFM phenotype, except one patient having short palm, small feet and duplication of genes from a portion of BTRC (exon 2–14, breakpoint at intron 1) to a portion of *FBXW4* (exon 9–2, breakpoint at intron 1) (Table S1, Patient 18). The inheritance was unknown. In four cases with classical SHFM phenotype, the duplicated segment contains genes from a portion of *LINC01514* (exon 3–6, breakpoint at intron 2) to a portion of *FBXW4* (exon 9–5, breakpoint at intron 4) having SHFM in the hands only (Table S1, Patient 19), while from LBX1 to a portion of *FBXW4* (exon 9–6, breakpoint at intron 5) (Table S1, Patient 20), from a portion of *LINC01514* (exon 4 to 6, breakpoint at intron 3) to a portion of *FBXW4* (exon 9–5, breakpoint at intron 4) (Table S1, Patient 21) and from *LBX1* to a portion of *FBXW4* (exon 9 to part of exon 1, breakpoint at exon 1) (Table S1, Patient 22) having SHFM in both hands and feet.

One patient with SHFM phenotype had a 1687 kb duplication at 10q23.33-q24.31, which contains 43 genes from a portion of *DNMBP* (exon 4 to 1, breakpoint at intron 4) to a portion of *FBXW4* (exon 9–6, breakpoint at intron 5) (Table S1, Patient 23). The inheritance was unknown. Four patients had the same 3112 kb duplicated region at 10q23.33-q25.1, which contains 82 genes from a portion of *DNMBP* (exon 4–1, breakpoint at intron 4) to a portion of *CNNM2* (exon 1–4, breakpoint at intron 4). SHFM phenotype was found in two patients (Table S1, Patient 24 and 25), but it was unknown in the other two cases. The inheritance was unknown for all four cases.

### 2.3. Breakpoint Mapping for Cases Without SHFM3 Phenotypes, but Gaining at the SHFM3 Locus

#### 2.3.1. PubMed Case

Using array comparative genomic hybridization, Fernández-Jaén *et al.* [12] in 2014 reported the first clinical case of a 126 kb microduplication at 10q24.31, affecting *LINC01514*, *LBX1*, *FLJ41350* and *AX747408*, in a 12 year-old girl with attention problems, dyspraxia, idiopathic congenital scoliosis, and marked atrophy of paravertebral muscles, and her paternal aunt with a severe and progressive myopathy (Table S1, Patient 41). *LBX1* plays a cardinal role in neuronal and muscular development in animal models, and has been reported as a candidate gene for idiopathic scoliosis, although its function in humans is unknown.

#### 2.3.2. DECIPHER Cases

One patient without classical SHFM phenotype, but having behavioural/psychiatric abnormality, constipation, deep-set eyes, delayed speech and language development, intellectual disability, macrocephaly, pectus excavatum, plagiocephaly, short stature and ventricular septal defect, had a 17,188 kb gain at 10q23.1–q26.11, which contains 243 genes from a portion of *IDE* (exon 14–1, breakpoint at intron 14) to *RNU6-53P* (Table S1, Patient 42). The inheritance was unknown.

### 2.4. Breakpoint Mapping for Cases Having Loss or Mutation at the SHFM3 Locus

#### 2.4.1. PubMed Cases

Vergult *et al.* [13] in 2013 screened a cohort of 54 patients with radial ray deficiencies (RRDs) for genomic aberrations using array comparative genomic hybridization. In eight of 54 cases, an aberration was detected, including an 80.2 kb microdeletion at 10q24.32 in a female with absence of right radius and thumb, shorter right ulna, left thenar hypoplasia and small apical ventricular septal defect at birth (Table S1, Patient 43). This deletion region contains a portion of *DPCD* (exon 2–6, breakpoint at intron 1), the entire *MIR3158-1*, *MIR3158-2*, and a portion of *FBXW4* (exon 9–4, breakpoint at intron 3). Molecular analysis of the parents revealed that the deletion was inherited from the unaffected mother. Since reduced penetrance, as also seen with the 10q24.3 duplications, may be possible in this case, the author postulated that the 10q24.3 deletion may result in RRDs by deletion of one or more specific regulatory regions. To test this hypothesis, six conserved noncoding elements (≥350 bp and sharing ≥90% identity with the mouse and/or ≥100 bp and sharing ≥70% identity with the frog and/or zebrafish) were selected in the deleted region on 10q24.3 based on literature review and on the use of the ECR (available online: http://ecrbrowser.dcode.org/), Ancora (available online: http://ancora.genereg.net/) and UCSC Genome Browser (available online: http://genome.ucsc.edu/), and were sequenced in the entire patient cohort. In a male patient with bilateral radius dysplasia, an A to G transition at genomic position 103380009 (GRCh 37, hg 19) (g.103380009A>G) was detected that was neither a single-nucleotide polymorphism nor present in a control cohort of 96 samples matched to the ethnicity of this patient or in the 1000 Genomes Project data (available online: http://browser.1000genomes.org). For this patient, no causal aberrations were detected using array CGH (Table S1, Patient 44). Unfortunately, parental DNA was unavailable. This A→G substitution could account for the RRDs seen in this patient, but functional studies need to be performed. Sequence analysis of the exons of *FBXW4* did not reveal causal mutations in the patient with the heterozygous *FBXW4* deletion or in any other patient of this cohort. This substitution is located at intron 6 of *FBXW4*. Although duplications in the 10q24.3 region result in split hand-foot malformations, this publication indicates that deletions may cause radial ray defects. However, the question of whether the deletion of the *FBXW4* gene itself or the presence of mutations in the flanking conserved noncoding regions are responsible for the limb defect remains open.

#### 2.4.2. DECIPHER Cases

Two male patients had loss or deletion at 10q24.31–q24.32. The first patient with short phalanx of finger, abnormality of the kidney, cleft palate, intellectual disability, microphthalmos, non-midline cleft lip, pulmonic stenosis, sensorineural hearing impairment, short stature, soft skin, truncal obesity and vesicoureteral reflux had a 546.42 kb loss at 10q24.31–q24.32, which contains 13 genes from *LINC01514* to a portion of *FBXW4* (exon 9–2, breakpoint at intron 1) (Table S1, Patient 45). The inheritance was unknown. The second patient with aortic dilatation, median cleft palate, Pierre-Robin sequence, renal hypoplasia and secundum atrial septal defect had a *de novo* 389.02 kb deletion at 10q24.31–q24.32, which contains genes from *BTRC* to a portion of *FBXW4* (exon 9–6, breakpoint at intron 5) (Table S1, Patient 46).

### 2.5. Discussion

Among the 48 cases presented above, 25 cases with microarray data have SHFM phenotypes (Table S1, Patients 1–25), 15 cases without microarray data have SHFM phenotypes (Table S1, Patients 26–40), two cases without SHFM phenotypes have gain at the SHFM3 locus (Table S1, Patients 41–42), four cases have loss or mutation at the SHFM3 locus (Table S1, Patients 43–46), and two cases with SHFM3 phenotypes and microarray data with duplication at the SHFM3 locus do not have breakpoint information (Table S1, Patient 47–48).

#### 2.5.1. Genotype and Phenotype Correlation

To explore the genotype and phenotype correlation, clinical presentation and genomic aberration in all cases were compiled in Table S1. In cases having microarray data, the shortest duplication is 126 kb at 10q24.31 resulting in the duplication of *LINC01514*, *LBX1*, *FLJ41350*, and *AX747408* from centromeric to telomeric direction in Patient 41 who does not have the SHFM phenotype, but has attention problems, dyspraxia, idiopathic congenital scoliosis, and marked atrophy of paravertebral muscles, and her paternal aunt has a severe and progressive myopathy. A 241.59 kb duplication at 10q24.31-q24.32 containing a portion of *BTRC* (exon 2–14, breakpoint at intron 1), the entire *POLL*, *DPCD*, *MIR3158-1*, *MIR3158-2*, and a portion of *FBXW4* (exon 9–2, breakpoint at intron 1) was found in Patient 18 who does not have classical SHFM phenotype, but has short palm and small feet, hypertelorism, intellectual disability, and obesity. The shortest duplication resulting in classical SHFM phenotype is 394 kb at 10q24.31–q24.32 containing *LBX1*, *FLJ41350*, *AX747408*, *BTRC*, *POLL*, and a portion of *DPCD* (exon 1, breakpoint at intron 1) from centromeric to telomeric direction in Patient 17. By real-time fluorescent quantitative PCR, the duplicate area encompassing the pathogenic genes was verified, and duplication in exon 9 of the nearby *FBXW4* gene was detected. In the majority of cases with SHFM phenotype, the duplication contains *LBX1*, *FLJ41350*, *AX747408*, *BTRC*, *POLL*, *DPCD*, *MIR3158-1*, *MIR3158-2*, and *FBXW4* or a portion of *FBXW4* from centromeric to telomeric direction. Some cases have a longer duplication region, such as a 1687 kb duplication at 10q23.33-q24.31 resulting in the duplication of 43 genes from a portion of *DNMBP* (exon 4–1, breakpoint at intron 4) to a portion of *FBXW4* (exon 9–6, breakpoint at intron 5) in Patient 23 who has the SHFM phenotype, and a 3112 kb duplication at 10q23.33-q25.1 resulting in the duplication of 82 genes from a portion of *DNMBP* (exon 4–1, breakpoint at intron 4) to a portion of *CNNM2* (exon 1–4, breakpoint at intron 4) in Patient 24 and 25, who have the SHFM phenotype. However, a 17,188 kb duplication at 10q23.1-q26.11 containing 243 genes from a portion of *IDE* (part of exon 15 to exon 1, breakpoint at exon 15) to *RNU6-53P* was found in Patient 42 who does not have the SHFM phenotype, but has behavioural/psychiatric abnormality, constipation, deeply set eyes, delayed speech and language development, intellectual disability, macrocephaly, pectus excavatum, plagiocephaly, short stature, and a ventricular septal defect. In SHFM cases with two discontinuous duplications at 10q24.31–q24.32 (Patient 12–15), the centromeric duplicated segment contains *LINC01514*, *LBX1*, *FLJ41350*, *AX747408*, and a portion of *BTRC* (exon 1, breakpoint at intron 1) from centromeric to telomeric direction, and the telomeric duplicated segment contains *POLL*, *DPCD*, *MIR3158-1*, *MIR3158-2*, and a portion of *FBXW4* (exon 9–2, breakpoint at intron 1) from centromeric to telomeric direction.

In SHFM cases without microarray data, the shortest duplication contains *BTRC*, *POLL*, *DPCD*, *MIR3158-1*, *MIR3158-2*, and a portion of *FBXW4* (exon 9–7), which was found in Patient 35 and 40. The majority of SHFM cases have duplication of *LBX1*, *BTRC*, *POLL*, *DPCD*, *MIR3158-1*, *MIR3158-2*, and *FBXW4* or a portion of *FBXW4*, which is the same as what was found in the cases having microarray data. In SHFM cases with two discontinuous duplications at 10q24, the centromeric duplicated segment containing *TLX1NB*, and a telomeric duplicated segment containing *LBX1*, *BTRC*, *POLL*, and a portion of *FBXW4* (exon 9–7) was found in Patient 26 and 32. Patient 29 has a centromeric duplicated segment containing *LBX1*, *BTRC*, *POLL*, and a portion of *FBXW4* (exon 9–3), and a telomeric duplicated segment containing *FGF8*, who has normal hands, but cleft and syndactyly of feet. Patients 30 and 31 have a centromeric duplicated segment containing *TLX1NB* and *TLX1* and a telomeric duplicated segment containing *BTRC*, *POLL*, *DPCD*, *MIR3158-1*, *MIR3158-2*, and a portion of *FBXW4* (exon 9–7). The phenotype of Patient 30 is unknown, but cleft hands and feet, oligodactyly and syndactyly were found in the affected family members. Patient 31 has duplication of a hand digit, triphalangeal thumb, and cleft, syndactyly and oligodactyly of feet.

The majority of the SHFM3 patients have anomalies in all four limbs, but Patient 19 has anomalies in the hands only, while Patients 29, 37 and 38 have anomalies in the feet only. In most cases with the SHFM phenotypes, the duplicated segment is at 10q24.31-q24.32 from the intergenic region centromeric to *LBX1* to the intergenic region telomeric to *FBXW4* (Figure S1), while the duplication in Patient 19 is from *LINC0154* (exon 3–6, breakpoint at intron 2) to *FBXW4* (exon 9–5, breakpoint at intron 4), Patient 29 has two duplicated segments, one containing genes from *LBX1* to *FBXW4* (exon 9–3) and the other containing *FGF8*, Patients 37 and 38 are from the same family having duplication from *LBX1* to *FBXW4* (exon 9–7).

Although there was no clear genotype and phenotype correlation found, the critical region responsible for the SHFM phenotype was identified and presented below.

#### 2.5.2. Critical Region Responsible for the SHFM Phenotype

To define the critical region resulting in the SHFM phenotype, the breakpoints of all cases having gain at the SHFM3 locus (Patient 1–42) were mapped, and the sizes of aberrations were compared, aligned and presented from centromeric to telomeric direction in Figure S1b. Patient 41 without the SHFM phenotype has duplication from *LINC01514* to *AX747408*, and Patient 18 without classical SHFM phenotype has duplication from a portion of *BTRC* (exon 2–14, breakpoint at intron 1) to a portion *FBXW4* (exon 9–2, breakpoint at intron 1); while Patients 12–15 with SHFM phenotypes have two duplicated segments, one from *LINC01514* to a portion of *BTRC* (exon 1, breakpoint at intron 1), and the other from *POLL* to a portion *FBXW4* (exon 9–2, breakpoint at intron 1). Therefore, the critical region responsible for the SHFM phenotypes is most likely located at exon 1 of *BTRC* gene, which is duplicated in all cases with SHFM phenotypes. Patient 42 has a 17188 kb duplication at 10q23.1–q26.11 containing 243 genes, including a portion of *IDE* (part of exon 15–1, breakpoint at exon 15) at the most centromeric region and *RNU6-53P* at the most telomeric region, which includes exon 1 of *BTRC*. However, Patient 42 does not have the SHFM phenotype. The reason is that large duplications may be associated with other phenotypes due to other pathomechanisms. There is only one copy number variation (CNV) overlapping *BTRC* exon 1 in the normal population (Figure S2), and it was observed as duplication in 4 out of 771 samples analyzed, located at 10q24.32 (chr10:103054982–103452645, hg 19) and containing genes from *BTRC* to a portion of *FBXW4* (exon 9–2) [14]. Therefore, the frequency of this duplication in the normal population is about 0.5%, which is very low. In addition, the four normal individuals having this duplication may be mosaic or have a more subtle SHFM phenotype, which remains unknown as detailed clinical information is not available.

## 3. Materials and Methods

### 3.1. Patient’s Phenotypes

**Figure 2:**
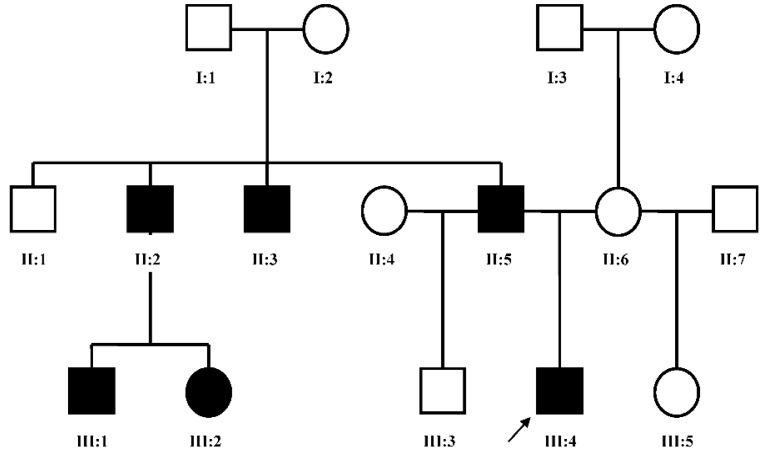
Pedigree of FH family: II:5 was examined clinically, and III:4 was examined clinically and genetically. Hollow box = normal male; black box = affected male; hollow circle = normal female; black circle = affected female; arrow = proband.

**Figure 3:**
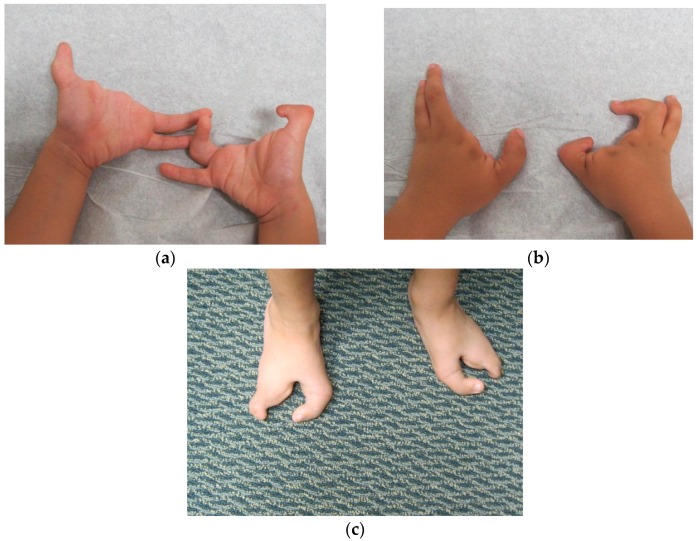
(**a**) FH palms; (**b**) FH hands; (**c**) FH feet.

**Figure 4:**
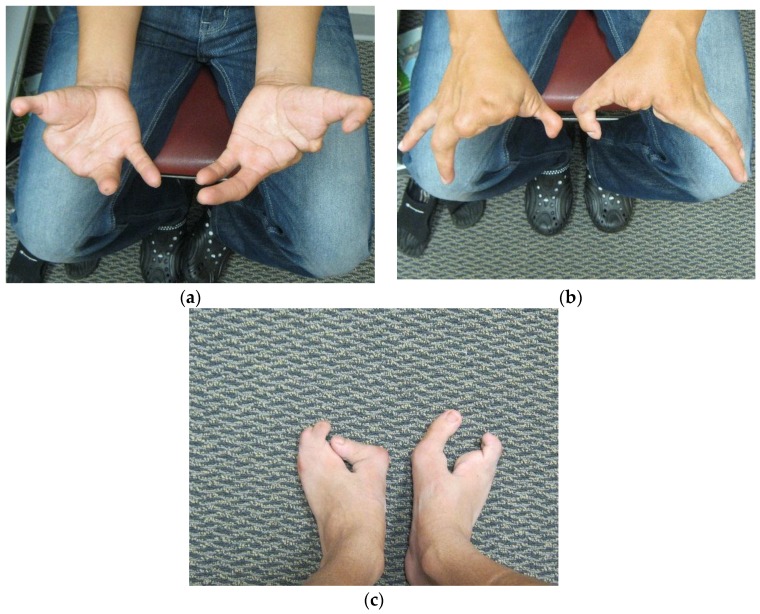
(**a**) Father’s palms; (**b**) Father’s hands; (**c**) Father’s feet.

Patient FH (Figure 2, III:4), a five year old male, presented to us for an initial syndromic evaluation. He was born by normal spontaneous vaginal delivery at term to non-consanguineous parents of Hispanic descent following an uncomplicated antenatal history. He had mild fine motor delay and otherwise normal growth and development. He was generally healthy, aside from exercise-induced asthma and recurrent reflux and constipation. On his left hand, FH was noted to have normal 4th and 5th digits, absent 2nd and 3rd digits, and camptodactyly of his thumb. On his right hand, he had a normal 5th digit, a 4th finger flexion deformity, absent 2nd and 3rd digits, and thumb camptodactyly (Figure 3a,b). He had pes planus, laterally deviated halluces, and absent 2nd, 3rd, and 4th toes bilaterally (Figure 3c). Other minor dysmorphic features included epicanthal folds, a bulbous nasal tip, anteverted nares, posteriorly rotated ears, a high arched palate, micrognathia, mild cubitus valgus, and a mild pectus excavatum deformity. His father (Figure 2, II:5) was observed to have similar hand and foot malformations. On his right hand, he had 5th finger campotodactyly, 4th finger camptodactyly and metacarpal aplasia, and rudimentary 2nd and 3rd digits. His right thumb was not fully formed, with anonychia and camptodacyly. On his left hand, he was noted to have mild 4th and 5th finger camptodactyly, rudimentary 2nd and 3rd digits, and thumb camptodactyly (Figure 4a,b). On his right foot, his hallux was curved inward, his 2nd and 3rd toes were missing with a cleft, and his 4th and 5th toes were fused together and curved inward. On his left foot, his hallux was curved inward perpendicular to his foot, his 2nd and 3rd toes were missing with a cleft, and his 4th and 5th toes were normal with a common base (Figure 4c). He was also noted to have retrognathia and several dental fillings. Detailed pedigree analysis revealed that similar hand and foot abnormalities were also present in two of FH’s three paternal uncles (Figure 2, II:2 and II:3), as well as two paternal first cousins (Figure 2, III:1 and III:2).

### 3.2. High Resolution Chromosome Analysis

Peripheral blood in heparin was obtained after informed consent, and high resolution chromosome analysis (band level > 650) was performed according to standard protocols.

### 3.3. Single Nucleotide Polymorphism (SNP) Microarray Analysis

A peripheral blood specimen was obtained after informed consent, and genomic DNA was extracted according to standard protocols. Single nucleotide polymorphism (SNP) microarray analysis was conducted using 6.0 SNP microarray from Affymetrix, which contains approximately 900,000 SNPs and 940,000 copy number probes throughout the human genome. The sample was analyzed at a resolution of 25 probes per 50 kb for the known microdeletion or microduplication syndromes and subtelomeric regions. The remaining genome was analyzed at a resolution of 50 probes per 200 kb. Affymetrix Genotyping Console was used to analyze the data. The practical resolution was set at ≥500 kb for duplications and ≥200 kb for deletions.

### 3.4. Database Search of Reported Cases

PubMed search for all available publications on SHFM3 was performed, as well as search on reported cases in the DECIPHER database using SHFM3 as the keyword. All PubMed cases after discovery of a tandem duplication at 10q24 in patients with SHFM3 in 2003 and all DECIPHER cases having clinical information were reviewed and presented below.

### 3.5. Mapping of Breakpoints

Breakpoints were mapped to the genomic location using available genomic information from the publications, and inputting them into UCSC Genome Browser (available online: http://genome.ucsc.edu/) with hg 18 or 19 assembly according to the assembly used in the publication to find the genetic coordinate for all cases reported in PubMed and DECIPHER databases.

## 4. Conclusions

Duplication of sequence in the exon 1 of *BTRC* is responsible for the formation of SHFM3 phenotype, which may be via cis-acting or trans-acting effects on genes or regulatory sequences involved in the limb development pathway.

The etiology of SHFM3 is due to disruption of the limb apical ectodermal ridge (AER). The SHFM3 locus at 10q24 is conserved in vertebrates from zebrafish to human, especially the region from *TLX1* to *FGF8*. AER is characterized by an unexpected regulatory complexity, with at least five distinct enhancers in the intragenic region of *FBXW4* (CE58, CE59, CE61 and CE66) and between *FBXW4* and *FGF8* (CE80) being autonomously active in the mouse embryo. In the human genome, the highest density of conserved noncoding elements is found in *BTRC*. Conserved noncoding elements can act as developmental enhancers with varying degrees of reproducibility. Extensive study of the regulatory architecture of this region showed that this region behaves as an integrated unit, a holo-enhancer: the internal organization of this intricate 200 kb interval has in itself an important role in integrating and filtering the activities of the multiple regulatory modules present within this region into a restricted tissue- and gene-specific output. This regulatory system may contribute to evolution of gene expression and account for the SHFM3 phenotypic consequences of genomic structural variants found in humans [15,16].

However, the above results were drawn from examination of the conserved noncoding elements. Future studies with an animal model containing sequence from the coding region, such as the sequence from exon 1 of *BTRC*, may explore its effect on AER and confirm our finding from mapping of the clinical SHFM3 cases. In addition, application of next generation sequencing, such as single molecular real-time (SMRT) sequencing, may identify the exact breakpoints at the nucleotide level to further refine the mapping of the critical region involved and the mechanism of rearrangements found in the SHFM3 cases, especially in those cases with two discontinuous duplications, as well as to verify the change of breakpoints if transmitted by the father. Furthermore, simultaneous epigenetic characterization during SMRT sequencing in cases with reduced penetrance may verify whether epigenetics is also involved in the pathogenesis of SHFM3, as has been found in the mouse model of this disorder [17].

## Supplementary Materials

The following are available online at http://www.mdpi.com/2076-3905/5/1/2/s1, Figure S1: Genetic landscape at SHFM3 locus, Figure S2: CNVs overlapping *BTRC* in normal population: Only variant esv2750847 (Pinto2007) shows as duplication overlapping exon 1 of *BTRC.* Table S1: Genotype and phenotype correlation.
