# Supplementary material for: Identification of Critical Region Responsible for Split Hand/Foot Malformation Type 3 (SHFM3) Phenotype through Systematic Review of Literature and Mapping of Breakpoints Using Microarray Data
Source: Microarrays (Basel). 2015 Dec 24;5(1):2. doi: 10.3390/microarrays5010002 (PMC5003447; doi:10.3390/microarrays5010002)
Supplement: Supplementary file 1 [file microarrays-05-00002-s001.pdf]

Supplementary Materials: Identification of Critical Region Responsible for Split Hand/Foot Malformation Type 3 (SHFM3) Phenotype through Systematic Review of Literature and Mapping of Breakpoints Using Microarray Data

Catherine F. Li, Katie Angione and Jeff M. Milunsky *

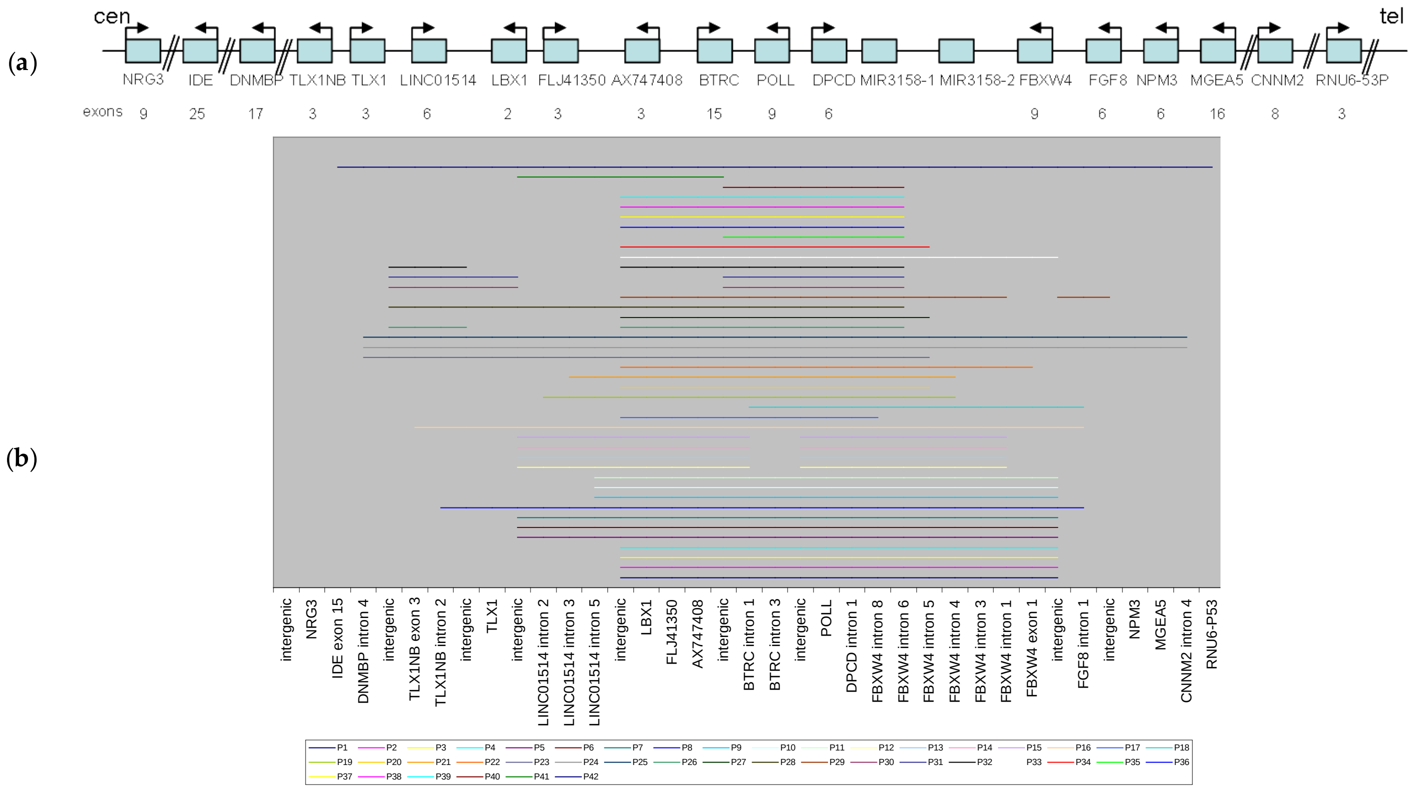

Figure S1. Genetic landscape at SHFM3 locus. (**a**) The order of genes at 10q21.1-qter from centromeric (cen) to telomeric (tel) direction; (**b**) Mapping of breakpoints in cases with gain at SHFM3 locus: In the majority of the cases with the SHFM phenotypes, the duplicated segment is at 10q24.31-q24.32 from the intergenic region centromeric to *LBX1* to the intergenic region telomeric to *FBXW4*. Patient 41 without the SHFM phenotype has duplication from *LINC01514* to *AX747408*, and Patient 18 without classical SHFM phenotype has duplication from a portion of *BTRC* (exon 2–14, breakpoint at intron 1) to a portion *FBXW4* (exon 9–2, breakpoint at intron 1); while Patient 12–15 with SHFM phenotypes have two duplicated segments, one from *LINC01514* to a portion of *BTRC* (exon 1,

breakpoint at intron 1), and the other from *POLL* to a portion *FBXW4* (exon 9–2, breakpoint at intron 1). Therefore, the minimum overlapping duplicated region among patients having the SHFM phenotypes is exon 1 of *BTRC* gene.

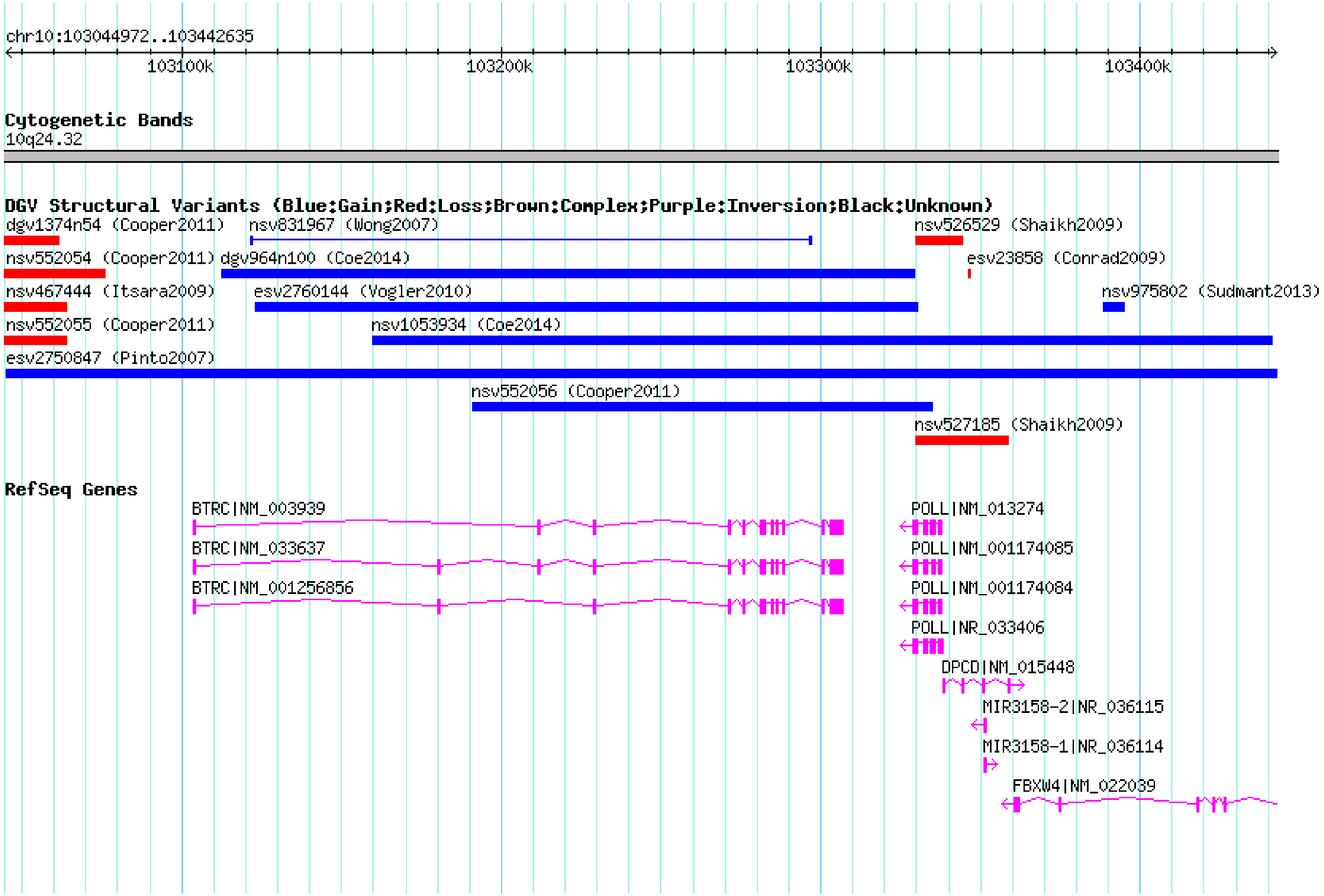

Figure S2. Copy number variations overlapping *BTRC* in normal population: Only variant esv2750847 (Pinto2007) shows as duplication overlapping exon 1 of *BTRC*.

Table S1. Genotype and phenotype correlation.

Patient ID	Limb Defect	Other Features	Methods	Genes in the Aberration	Reference
1	Left hand: normal 4th and 5th digits, absent 2nd and 3rd digits, and camptodactyly of thumb<br>Right hand: normal 5th digit, a 4th finger flexion deformity, absent 2nd and 3rd digits, and thumb camptodactyly<br>Lower limb: pes planus, laterally deviated halluces, and absent 2nd, 3rd, and 4th toes bilaterally	Epicanthal folds, a bulbous nasal tip, anteverted nares, posteriorly rotated ears, a high arched palate, micrognathia, mild cubitus valgus, and a mild pectus excavatum deformity	6.0 SNP microarray: 514 Kb duplication at 10q24.31-10q24.32 (chr10:102962134-103476346, hg 19)	From *LBX1* to *FBXW4*	Proband in this study
2	Upper limbs: absent radii, hypoplastic curved ulnae, rudimentary carpal bones, absence of other bones of the hands<br>Lower limbs: normal long bones, bilateral synostosis of rudimentary calcaneus and talus, absent tarsal bones, one short and dysplastic tubular bone of both feet	Maxillary hypoplasia, micrognathia, high arched palate, severe myopia, low set ears, recurrent otitis in childhood, bilateral conductive hearing loss, oligomega-nephronia, moderate intellectual disability, undescended left testis	Array comparative genomic hybridization: 532.77 kb triplication at 10q24(chr10:102965299-103498069, hg18); confirmed by FISH and qPCR	Same as the above	F1 C2755 (brother of C224256)
3	Upper limbs: deformation and bowing of distal radii, absent carpal bones, missing 1st right and 2nd right and 1st left metacarpals, one rudimentary dysmorphic bone between 3rd and 4th metacarpals, only one 5th finger with three phalanges<br>Lower limbs: normal long bones, talus, calcaneus, naviculare and cuboideum, rudimentary 1st metatarsal, missing 2-4 metatarsals, 5th metatarsal with large base and one two-phalangeal digit	Maxillary hypoplasia, micrognathia, high arched palate, low set ears, borderline intellectual disability	Array comparative genomic hybridization: 532.77 kb duplication at 10q24(chr10:102965299-103498069, hg18); confirmed by FISH and qPCR	Same as the above	F1 C224256 (sister of C224256)
4	Upper limbs: normal long bones, two metacarpals, one postaxial (5th) finger with two phalanges<br>Lower limbs: normal long bones, one metatarsal, one postaxial digit with one phalanx	Maxillary hypoplasia, micrognathia, cleft palate, nystagmus, severe myopia, low set ears, recurrent otitis, bilateral conductive hearing loss, renal hypoplasia	Array comparative genomic hybridization: 528.72 kb duplication at 10q24(chr10:102969344-103498069, hg18); confirmed by qPCR	Same as the above	F2 C211000

Table S1. *Cont.*

Patient ID	Limb Defect	Other Features	Methods	Genes in the Aberration	Reference
5	Normal	None	Array comparative genomic hybridization: somatic mosaic for 597.29 kb duplication at 10q24(chr10:102900770-103498069, hg18); confirmed by FISH and qPCR	From *LINC01514* to *FBXW4*	F3 C369371 (mother of C369373 and C369374)
6	Upper limbs: classical SHFM<br>Lower limbs: classical SHFM	None	Array comparative genomic hybridization: 597.29 kb duplication at 10q24(chr10:102900770-103498069, hg18); confirmed by qPCR	Same as the above	F3 C369373 (sister of C369374)
7	Upper limbs: normal long bones, four metacarpals, one postaxial (5th) finger with three phalanges, one phalanx crossing between 4th and 5th metacarpals and one rudimentary bone between 3rd and 4th metacarpals<br>Lower limbs: classical SHFM	Maxillary hypoplasia, micrognathia, low set ears, recurrent otitis, bilateral mixed conductive-sensorineural hearing loss	Same as the above	Same as the above	F3 C369374 (brother of C369373)
8	Upper limbs: classical SHFM<br>Lower limbs: classical SHFM	renal hypoplasia	Array comparative genomic hybridization: 658.43 kb duplication at 10q24(chr10:102870153-103528589, hg18); confirmed by qPCR	From a portion of *TLX1NB* (exon 2 to 1, breakpoint at intron 2) to a portion of *FGF8* (exon 6 to 2, breakpoint at intron 1)	F4 C365650
9	Upper limbs: classical SHFM<br>Lower limbs: classical SHFM	Long and narrow face, mild facial asymmetry, micrognathia, high palate, bilateral congenital cataracts, hypertension	Array comparative genomic hybridization: 539 kb duplication at 10q24.31–q24.32(chr10:102942925-103481863, hg18); confirmed by qPCR	From a portion of *LINC01514* (exon 6, breakpoint at intron 5) to *FBXW4*	Patient 1 (older brother of Patient 2)
10	Upper limbs: monodactyly of the right hand, bidactyly of the left hand<br>Lower limbs: monodactyly of both feet	Mild myopia, sensorineural hearing loss	Same as the above	Same as the above	Patient 2 (younger brother of Patient 1)
11	Normal	None	qPCR showed that mother's DNA carries the duplication in 20% of blood lymphocytes	Same as the above	Mother of Patient 1 and Patient 2

Table S1. *Cont.*

Patient ID	Limb Defect	Other Features	Methods	Genes in the Aberration	Reference
12	Left upper limb: proximal placed thumb; triphalangeal thumb; camptodactyly of 1st and 2nd finger; 3/4 syndactyly<br>Right upper limb: proximal placed thumb; camptodactyly of thumb; triphalangeal thumb; missing 2nd finger; 3/4 cutaneous syndactyly<br>Left lower limb: camptodactyly of 1st toe; absence of 2nd toe; transversely placed proximal phalange of 3rd toe; deficiency of middle, distal phalanges of 3rd toe; cleft foot<br>Right lower limb: central cleft between 1/2 toe syndactyly and 3/4 toe syndactyly; absence of middle and distal phalanges of 3rd toe; misplaced proximal phalanx of 2nd toe	None	Array comparative genomic hybridization: duplication at 10q24.31–q24.32 containing two discontinuous DNA fragments. The centromeric duplicated segment is 257 kb (chr10:102911736-103169477, hg 19); The telomeric duplication is 114 kb (chr10:103334900-103449414, hg 19). Confirmed by qPCR.	Centromeric duplicated segment: from *LINC01514* to a portion of *BTRC* (exon 1, breakpoint at intron 1); Telomeric duplicated segment: from *POLL* to a portion of *FBXW4* (exon 9 to 2, breakpoint at intron 1)	II:5 (father of III:9 and III:10)
13	Left upper limb: Misplaced and hypoplastic 1st metacarpal; missing thumb; absence of 2nd finger; missing middle and distal phalanges of 3rd finger; osteal fusion between 4th finger and transversely placed proximal phalanges of 3rd finger<br>Right upper limb: Absence of 1st ray and thumb; hypoplasia of 2nd metacarpal; missing 2nd finger; absence of middle, distal phalanges of 3rd finger; nail aplasia of 4th finger<br>Left lower limb: Median cleft due to absence of 2nd toe; missing distal phalanx of 3rd toe; transversely placed proximal phalanx of 3rd toe; 3/4 toe syndactyly; abnormal metatarsals; clinodactyly of 4th toe<br>Right lower limb: Absence of 2nd and 3rd toes; hypoplasia of 2nd metatarsus; cleft foot	None	Array comparative genomic hybridization: duplication at 10q24.31–q24.32 containing two discontinuous DNA fragments. The centromeric duplicated segment is 259 kb (chr10:102911736-103170849, hg 19); The telomeric duplication is 125 kb (chr10:103324414–103449414, hg 19). Confirmed by qPCR.	Same as the above	III:9 (brother of III:10)

Table S1. *Cont.*

Patient ID	Limb Defect	Other Features	Methods	Genes in the Aberration	Reference
14	Left upper limb: Missing 1st ray and thumb; absence of the middle, distal phalanges of 2nd finger; 3/4 syndactyly Right upper limb: Absence of 1st ray and thumb; deficiency of middle, distal phalanx of 2nd finger; 3/4 syndactyly Left lower limb: Central cleft due to absence of 2nd toe; hypoplasia of 2nd metatarsus; missing distal phalanx of 3rd toe; 3/4 toe syndactyly; cleft foot Right lower limb: Central cleft due to absence of 2nd and 3rd toes; hypoplasia of 2nd metatarsus	None	Array comparative genomic hybridization: duplication at 10q24.31–q24.32 containing two discontinuous DNA fragments. The centromeric duplicated segment is 257 kb (chr10:102911736-103169477, hg 19); The telomeric duplication is 116 kb (chr10:103332499-103449414, hg 19). Confirmed by qPCR.	Same as the above	III:10 (sister of III:9)
15	Left upper limb: Duplicated distal phalanx of 4th finger; missing 1st metacarpal; proximal placed thumb remnant; missing middle and distal phalanges of 2nd, 3rd fingers; 3/4 cutaneous syndactyly Right upper limb: Absence of 1st metacarpal and thumb; missing middle, distal phalanges of 2nd and 3rd finger; cutaneous 3/4 syndactyly Left lower limb: 1/2 toe syndactyly; extra proximal phalanx of 2nd toe; 3/4 toe syndactyly; median cleft Right lower limb: Cleft due to absence of 2nd toe; 3/4 toe syndactly	None	Array comparative genomic hybridization: duplication at 10q24.31-q24.32 containing two discontinuous DNA fragments. The centromeric duplicated segment is 247 kb (chr10:102911736-103159334, hg 19); the telomeric duplication is 114 kb (chr10:103334900-103449414, hg 19). Confirmed by qPCR.	Same as the above	IV:3 (son of III:9)
16	Anomalies of all four limbs, consisting of a combination of central reduction defects, preaxial polydactyly, triphalangeal thumb, and central polydactyly, with the hands more severely affected than the feet	Inverted nipples	Array comparative genomic hybridization: 600 kb duplication at 10q24.31q24.32 (chr10:102840000-103440000, hg 18)	From a portion of *TLX1NB* (part of exon 3 to 1, breakpoint at exon 3) to a portion of *FBXW4* (exon 9 to 2, breakpoint at intron 1)	Proband

Table S1. *Cont.*

Patient ID	Limb Defect	Other Features	Methods	Genes in the Aberration	Reference
17	Split-hand/split-foot malformation	None	Genome-wide copy number variants SNP microarray: 394 kb duplication at 10q24.31–24.32 (chr10:102955122-103348688, hg 19); Quantitative PCR: duplication in exon 9 of FBXW4 gene	From *LBX1* to a portion of *DPCD* (exon 1, breakpoint at intron 1)	Proband
18	Short palm, small feet	Hypertelorism, intellectual disability, and obesity	Array comparative genomic hybridization: 241.59 kb duplication at 10q24.31-q24.32(chr10: 103205784-103447377, hg 19)	From a portion of *BTRC* (exon 2 to 14, breakpoint at intron 1) to a portion of *FBXW4* (exon 9 to 2, breakpoint at intron 1)	ID 256278 DECIPHER
19	Aplasia/hypoplasia of the thumb, and aplasia of the fingers	None	Array comparative genomic hybridization: 494.54 kb duplication at 10q24.31-q24.32(chr10:102936862-103431400, hg 19)	From a portion of *LINC01514* (exon 3 to 6, breakpoint at intron 2) to a portion of *FBXW4* (exon 9 to 5, breakpoint at intron 4)	ID 259243 DECIPHER
20	Abnormality of the foot and the hand	None	Array comparative genomic hybridization: 438.20 kb duplication at 10q24.31-q24.32(chr10:102969339-103407534, hg 19)	From *LBX1* to a portion of *FBXW4* (exon 9 to 6, breakpoint at intron 5)	ID 261018 DECIPHER
21	Ectrodactyly (feet and hands)	Bicuspid aortic valve	Array comparative genomic hybridization: 480 kb gain at 10q24.31-q24.32(chr10:102948861-103428862, hg 19)	From a portion of *LINC01514* (exon 4 to 6, breakpoint at intron 3) to a portion of *FBXW4* (exon 9 to 5, breakpoint at intron 4)	ID 261574 DECIPHER
22	Ectrodactyly (feet and hands)	None	Array comparative genomic hybridization: 485.15 kb duplication at 10q24.31-q24.32(chr10:102969369-103454514, hg 19)	From *LBX1* to a portion of *FBXW4* (exon 9 to part of exon 1, breakpoint at exon 1)	ID 283763 DECIPHER

Table S1. *Cont.*

Patient ID	Limb Defect	Other Features	Methods	Genes in the Aberration	Reference
23	Absent hand, lower limb malformation	Abnormality of the kidney, abnormality of the temporomandibular joint, hearing impairment, high palate, intellectual disability, micrognathia, midface retrusion, narrow palate, proportionate short stature, and sparse hair	Array comparative genomic hybridization: 1687 kb duplication at 10q23.33-q24.31(chr10:101711724-103398603, hg 19)	43 genes from a portion of *DNMBP* (exon 4 to 1, breakpoint at intron 4) to a portion of *FBXW4* (exon 9 to 6, breakpoint at intron 5)	ID 249629 DECIPHER
24	Absent toe, aplasia of the fingers	Abnormality of the temperomandibular joint, high palate, micrognathia, midface retrusion, nephrolithiasis, proportionate short stature, and secondary amenorrhea	Array comparative genomic hybridization: 3112 kb duplicated region at 10q23.33-q25.1 (chr10:101711724-104823513, hg 19)	82 genes from a portion of *DNMBP* (exon 4 to 1, breakpoint at intron 4) to a portion of *CNNM2* (exon 1 to 4, breakpoint at intron 4)	ID 249450 DECIPHER
25	Ectrodactyly (hands)	Cleft palate, micrognathia, and myopia	Same as the above	Same as the above	ID 249514 DECIPHER
26	Hands: cleft, syndactyly<br>Feet: unknown	None	Southern, pulsed field gel electrophoresis, dosage analyses	Centromeric duplicated segment: *TLX1NB*; Telomeric duplicated segment: from *LBX1* to a portion of *FBXW4* (exon 9 to 7)	Patient DF
27	Hands: syndactyly<br>Feet: cleft, syndactyly, oligodactyly	None	Southern, pulsed field gel electrophoresis, dosage analyses	*LBX1* to *FBXW4* (exon 9 to 6)	Patient AC
28	Hands: oligodactyly, syndactyly<br>Feet: cleft, syndactyly, oligodactyly	None	Southern, pulsed field gel electrophoresis, dosage analyses; FISH, quantitative PCR	From *TLX1NB* to a portion of *FBXW4* (exon 9 to 7)	Patient LS
29	Hands: Normal<br>Feet: cleft, syndactyly	None	Southern, pulsed field gel electrophoresis, dosage analyses	Centromeric duplicated segment: from *LBX1* to a portion of *FBXW4* (exon 9 to 3); Telomeric duplicated segment: FGF8	Patient VB

Table S1. *Cont.*

Patient ID	Limb Defect	Other Features	Methods	Genes in the Aberration	Reference
30	Unknown, but cleft hands/feet, oligodactyly, syndactyly in the affected family members	None	Southern, pulsed field gel electrophoresis, dosage analyses	Centromeric duplicated segment: from *TLX1NB* to *TLX1*; Telomeric duplicated segment: from *BTRC* to a portion of *FBXW4* (exon 9 to 7)	Patient DH
31	Hands: duplication of a digit, triphalangeal thumb<br>Feet: cleft, syndactyly, oligodactyly	None	Southern, pulsed field gel electrophoresis, dosage analyses	Same as the above	Patient TU
32	Hands: cleft, syndactyly, oligodactyly<br>Feet: cleft, syndactyly, oligodactyly	Medulloblastoma	Southern, pulsed field gel electrophoresis, dosage analyses	Centromeric duplicated segment: *TLX1NB*; Telomeric duplicated segment: from *LBX1* to a portion of *FBXW4* (exon 9 to 7)	Patient RH
33	Hands: cleft, syndactyly, oligodactyly<br>Feet: cleft, syndactyly, oligodactyly	None	Southern blot	From *LBX1* to *FBXW4*	Patient AY
34	Hands: cleft, oligodactyly<br>Feet: cleft, oligodactyly	None	Southern blot	From *LBX1* to a portion of *FBXW4* (exon 9 to 6)	Patient YI
35	Hands: cleft, syndactyly, oligodactyly<br>Feet: cleft, syndactyly, oligodactyly	None	FISH, quantitative PCR	From *BTRC* to a portion of *FBXW4* (exon 9 to 7)	RK040 II-4 (father of RK040 III-4)
36	Hands: cleft, syndactyly, oligodactyly<br>Feet: cleft, syndactyly, oligodactyly	None	FISH, quantitative PCR	From *LBX1* to a portion of *FBXW4* (exon 9 to 7)	RK040 III-4 (daughter of RK040 II-4)
37	Hands: Normal<br>Feet: bilateral split-foot	None	FISH, quantitative PCR	From *LBX1* to a portion of *FBXW4* (exon 9 to 7)	EC001 III-3 (brother of EC001 III-11)
38	Hands: Normal<br>Feet: bilateral split-foot	None	FISH, quantitative PCR	Same as the above	EC001 III-11 (sister of EC001 III-3)
39	Hands: absent fingers 1–3 with syndactyly of fingers 4–5 bilaterally<br>Feet: bilateral split-foot	None	FISH, quantitative PCR	Same as the above	K6421 Case 1

Table S1. *Cont.*

Patient ID	Limb Defect	Other Features	Methods	Genes in the Aberration	Reference
40	Hands: triphalangeal thumb on left; bifid thumb on right with syndactyly of index finger, hypoplastic 3rd nail, and cleft separating 2nd–3rd fingers Feet: bilateral split-foot	None	FISH, quantitative PCR	From *BTRC* to a portion of *FBXW4* (exon 9 to 7)	K6459 Case 2
41	None	Attention problems, dyspraxia, idiopathic congenital scoliosis, and marked hypotrophy of paravertebral muscles	Array comparative genomic hybridization: 126 kb microduplication at 10q24.31 (chr10:102927883-103053612, hg19)	From *LINC01514* to *AX747408*	Proband
42	None	Behavioral/psychiatric abnormality, constipation, deep-set eyes, delayed speech and language development, intellectual disability, macrocephaly, pectus excavatum, plagiocephaly, short stature, and ventricular septal defect	Array comparative genomic hybridization: 17188 kb gain at 10q23.1-q26.11(chr10:94214092-111401818, hg 19)	243 genes from a portion of *IDE* (part of exon 15 to 1, breakpoint at exon 15) to *RNU6-53P*	ID 2578 DECIPHER
43	Radial ray defect with absence of right radius and thumb, shorter right ulna, left thenar hypoplasia	Small apical ventricular septal defect	Array comparative genomic hybridization: 80.2 kb microdeletion at 10q24.32(chr10:103352980-103433225, hg 19)	From a portion of *DPCD* (exon 2-6, breakpoint at intron 1 to a portion of *FBXW4* (exon 9 to 4, breakpoint at intron 3)	Patient 7
44	Bilateral radial dysplasia	None	Array comparative genomic hybridization: Normal, but an A to G transition on genomic position chr10:103380009 (GRCh 37, hg 19) (g.103380009A>G)	This substitution is located at intron 6 of *FBXW4*	Patient 9

Table S1. *Cont.*

Patient ID	Limb Defect	Other Features	Methods	Genes in the Aberration	Reference
45	Short phalanx of finger	Abnormality of the kidney, cleft palate, intellectual disability, microphthalmos, non-midline cleft lip, pulmonic stenosis, sensorineural hearing impairment, short stature, soft skin, truncal obesity, and vesicoureteral reflux	Array comparative genomic hybridization: 546.42 kb loss at 10q24.31-q24.32(chr10:102905119-103451543, hg 19)	From *LINC01514* to a portion of *FBXW4* (exon 9 to 2, breakpoint at intron 1)	ID 4626 DECIPHER
46	None	Aortic dilatation, median cleft palate, Pierre-Robin sequence, renal hypoplasia, and secundum atrial septal defect	Array comparative genomic hybridization: 389.02 kb deletion at 10q24.31-q24.32(chr10:103000864-103389882, hg 19)	From *BTRC* to a portion of *FBXW4* (exon 9 to 6, breakpoint at intron 5)	ID 290836 DECIPHER
47	Split-hand and split-feet	Unknown	6.0 SNP microarray: 560 kb duplication at 10q24; confirmed by qPCR.	Unknown	Proband
48	Split-hand and split-feet	Unknown	Array comparative genomic hybridization: 662.3 kb duplication at 10q24.31-q24.32	Unknown	Proband
